# Early Tracheostomy May Reduce the Length of Hospital Stay

**DOI:** 10.1155/2023/8456673

**Published:** 2023-08-18

**Authors:** Fernanda Kazmierski Morakami, Ana Luiza Mezzaroba, Alexandre Sanches Larangeira, Lucienne Tibery Queiroz Cardoso, Carlos Augusto Marçal Camillo, Cintia Magalhães Carvalho Grion

**Affiliations:** Universidade Estadual de Londrina, Rua Robert Koch, n° 60, Vila Operária, Londrina, Paraná, Brazil

## Abstract

**Introduction:**

There is evidence that prolonged invasive mechanical ventilation has negative consequences for critically ill patients and that performing tracheostomy (TQT) could help to reduce these consequences. The ideal period for performing TQT is still not clear in the literature since few studies have compared clinical aspects between patients undergoing early or late TQT.

**Objective:**

To compare the mortality rate, length of stay in the intensive care unit, length of hospital stay, and number of days free of mechanical ventilation in patients undergoing TQT before or after ten days of orotracheal intubation.

**Methods:**

A retrospective cohort study carried out by collecting data from patients admitted to an intensive care unit between January 2008 and December 2017. Patients who underwent TQT were divided into an early TQT group (i.e., time to TQT ≤ 10 days) or late TQT (i.e., time to TQT > 10 days) and the clinical outcomes of the two groups were compared.

**Results:**

Patients in the early TQT group had a shorter ICU stay than the late TQT group (19 ± 16 vs. 32 ± 22 days, *p* < 0.001), a shorter stay in the hospital (42 ± 32 vs. 52 ± 50 days, *p* < 0.001), a shorter duration of mechanical ventilation (17 ± 14 vs. 30 ± 18 days, *p* < 0.001), and a higher proportion of survivors in the ICU outcome (57% vs. 46%, *p* < 0.001).

**Conclusion:**

Tracheostomy performed within 10 days of mechanical ventilation provides several benefits to the patient and should be considered by the multidisciplinary team as a part of their clinical practice.

## 1. Introduction

Tracheostomy (TQT) is a surgical procedure commonly performed in critically ill patients admitted to intensive care units (ICUs) [1]. The literature is still unclear about the superiority of the surgical technique, patient eligibility, indication for performing invasive mechanical ventilation (IMV), and decannulation based on the duration [2].

IMV is considered prolonged based on the patient's dependence on ventilatory support for a time longer than 6 hours a day and for a time longer than 21 days [3]. The use of IMV has the following possible consequences: pneumonia associated with IMV [4], diaphragmatic dysfunction [5], critically ill polyneuropathy, longer ICU and hospital stays, and higher healthcare costs [6–8]. In addition, performing TQT has been used as a strategy to shorten IMV time, and there is evidence that it is an independent predictor for this outcome, with shorter ICU and hospital stays and lower healthcare costs [8].

Despite being widely used, the ideal time for performing a tracheostomy is still much discussed in the scientific literature, with recommendations that vary between the most diverse periods of use of IMV, especially when considering definitions such as early and late tracheostomy [8–10]. Andriolo et al. in Cochrane review [9] used an arbitrary cut-off point of ten days since the patient is usually submitted to tracheostomy by the fourteenth day of orotracheal intubation. However, there is still divergence in the literature regarding the association between early (≤10 days) and late (>10 days) tracheostomy in relation to mortality, length of stay in the ICU, length of hospital stay, and days free of mechanical ventilation. The current study aimed to compare these clinical outcomes in critically ill patients at a tertiary hospital over a period of ten years.

## 2. Methods

This is a retrospective study carried out from January 2008 to December 2017 at the University Hospital of the State University of Londrina, Brazil. Patients admitted to the ICU during the study period who underwent tracheostomy were included, and patients under 18 years of age and/or those who had incomplete data in their medical records were excluded. The following data were collected for the patients: anthropometric data, diagnosis of admission to the ICU, date and outcome at ICU discharge, date and outcome at hospital discharge, calculation of the Charlson comorbidity index [11], date of tracheostomy, time of IMV before and after tracheostomy, length of stay in the ICU, and acute physiology and chronic health evaluation II (APACHE II) [12] and sequential organ failure assessment (SOFA) [13] scores calculated at the time of admission to the ICU. The patients were divided into two groups, according to the cut-off point used for classification; early TQT (ET) and late TQT (LT) were 10 days of orotracheal intubation, counted from the date it occurred. Patients were followed up until hospital discharge or death, and data were obtained from the patient's medical record and the institution's electronic database. The day of the tracheostomy was determined by the intensivist responsible for the patient and according to the dynamics of the service. The study was approved by the Institutional Ethics and Research Committee (n° 225/23124.2016.14), which also waived the free and informed consent form, with the researcher committing to ethical provisos and secrecy.

## 3. Statistical Analysis

SAS (statistical analysis system) software was used for data analysis. For analysis of the distribution of normality of the data, the Shapiro–Wilk test was used. Results are described as the mean and standard deviation (SD) or the median and interquartile range (25%–75%). The chi-square test was used to compare categorical variables and the Mann–Whitney test and the Wilcoxon sample test to compare the early TQT and late TQT groups. To assess the risk of mortality using possible predictors, univariate and multivariate logistic regression models were used. Finally, to assess the discriminative power of the ten-day cut-off point for hospital and ICU mortality, the area under the curve in the receiver-operating characteristic (ROC) analysis was used, and values ≥0.7 were considered adequate. The level of statistical significance adopted was considered *p* < 0.05.

## 4. Results

A total of 1080 patients were included in the sample, admitted to the ICU and who underwent tracheostomy during the ten-year period of the study. Five patients were excluded due to the unavailability of complete data for analysis; so, 1075 patients were analyzed in the present study. The early tracheostomy (ET) group included 552 (51%) patients, with 366 men and a mean age 58 ± 18 years. The late tracheostomy (LT) group included 523 patients, with 317 men and a mean age of 56 ± 20 years (Table 1). Regarding the scores in the assessment tools, patients in the LT group presented more organ dysfunction (*p* < 0.0001) in the SOFA index and a greater number of comorbidities (*p* < 0.003) compared to the group with ET; however, no differences were observed in relation to the probability of death, measured by the APACHE II, when compared to the ET group (Table 2). When the groups are compared for the mean PaO_2_/FiO_2_ ratio to evaluate the severity of the pulmonary impairment, a lower PaO_2_/FiO_2_ ratio was observed in the ET group (*p* < 0.03) (Table 2).

Patients in the ET group spent less time under IMV, both when analyzing the days on IMV and the days free from mechanical ventilation. The ET group had a shorter length of stay in the ICU (*p* < 0.001) and in the hospital (*p* < 0.001) than the LT group (Table 2) with a difference of 13 days for ICU and 10 days for hospitalization. A higher proportion of survivors at ICU discharge was observed among patients in the ET group compared to the LT group (57% vs. 46%, *p* < 0.001) but no difference was found regarding the outcome of survival at hospital discharge between the two groups (*p* > 0.05).

**Figure 1:**
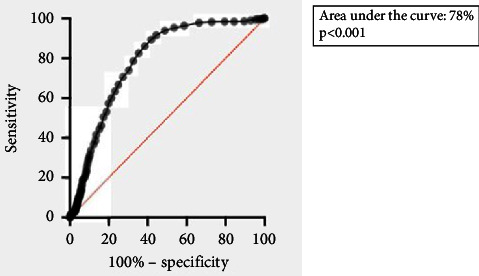
Analysis of the area under the ROC curve for early tracheostomy and length of stay in the ICU.

No discriminatory power was observed between the time of tracheostomy for mortality in the ICU or in the hospital, but for the length of stay in the ICU, the area under the curve was 78%, with *p* < 0.001 (Figure 1).

## 5. Discussion

This study contributes to the scientific literature in recommending early tracheostomy in critically ill patients, under prolonged IMV, since benefits were observed when the TQT was performed before 10 days of orotracheal intubation. The clinical characteristics of patients in the early (ET) and late (LT) tracheostomy groups differ in terms of organ dysfunction and the number of comorbidities associated with the main diagnosis, with the ET group presenting a lower number of comorbidities and also a lower degree of organic dysfunction according to the evaluation of the SOFA instrument. These data partially corroborate the literature since studies report the benefits of early tracheostomy, regardless of the difference in the scores presented between the studied groups [14–16]. When evaluating the difference in hospital outcomes, no differences were found in hospital mortality between the groups and submitting the patient to early tracheostomy did not influence the hospital survival outcome [17–19]. Despite mortality being an outcome of great importance in clinical practice and the lack of difference between the early and late groups, the benefits of early tracheostomy should still be considered at the time of clinical decision, since it is necessary to evaluate health costs when variables such as days spent in the ICU and in the hospital are analyzed.

A Cochrane systematic review, published in 2015, by Andriolo et al. [9], reported an analysis adopting the arbitrary cut-off point of the early and late TQT of 10 days, as well as in the present study. In that review, the authors demonstrated that patients undergoing early tracheostomy remain on IMV for a shorter time (both for IMV days and IMV-free days) and have a shorter ICU stay and hospital stay, results that were also found in this study. Although there is a statistical difference between the groups for the PaO_2_/FiO_2_ ratio, it is not a clinically relevant difference as they reflect mild hypoxemia in both groups. Thus, the current study corroborates the scientific literature regarding the recommendation of early tracheostomy in critically ill patients, a topic that still needs to be further studied.

## 6. Strengths and Limitations

Despite the retrospective characteristic, the present study includes a large number of patients and has a long duration, contributing to the current literature regarding the ideal time to perform a tracheostomy.

## 7. Conclusion

Performing a tracheostomy within 10 days of orotracheal intubation in critically ill patients provides less time of use of invasive mechanical ventilation and a shorter length of stay in the intensive care unit and hospital, despite not showing differences in the in-hospital mortality rate.

## Figures and Tables

**Table 1:** Clinical characteristics of the sample and comparison of early and late tracheostomy groups.

	Total (*n* = 1075)	Early tracheostomy (*n* = 552)	Late tracheostomy (*n* = 523)	*p* value
Sex (F/M)	392/683	186/366	206/317	>0.05
Age (years)	57 ± 19	58 ± 18	56 ± 20	>0.01
Performing a tracheostomy (days)	11 ± 16	5 ± 3	18 ± 22	<0.0001
SOFA	9 [7–12]	9 [7–11]	10 [8–12]	<0.0001
APACHE II	25 [20–32]	25 [20–31]	26 [20–33]	>0.05
Number of comorbidities	2 [0–3]	5 [1–7]	7 [2–7]	<0.003

F: female; M: male; SOFA: sequential organ failure assessment; APACHE: acute physiology and chronic health disease classification system.

**Table 2:** Comparison of the length of stay and outcomes of the early and late tracheostomy groups.

	Total (*n* = 1075)	Early tracheostomy (*n* = 552)	Late tracheostomy (*n* = 523)	*p* value
ICU length of stay (days)^*∗*^	25 ± 20	19 ± 16	32 ± 22	<0.0001
Hospital length of stay (days)^*∗*^	47 ± 42	42 ± 32	52 ± 50	<0.0001
Time on mechanical ventilation (days)^*∗*^	23 ± 18	17 ± 14	30 ± 18	<0.0001
Time off mechanical ventilation (days)^*∗*^	23 ± 38	25 ± 28	21 ± 47	<0.0002
ICU outcome (survival/death)	555/520	57%/43%	46%/54%	<0.0006
Hospital outcome (survival/death)	298/777	28%/72%	28%/72%	>0.05
PaO_2_/FiO_2_ ratio^*∗∗*^	248 [183–324]	242 [175–320]	255 [192–322]	<0.03

ICU: intensive care unit. PaO_2_: partial pressure of oxygen in arterial blood. FiO_2_: fraction of inspiratory oxygen concentration: ^*∗*^mean ± standard deviation; ^*∗∗*^median (interquartile range).

## Data Availability

The data used to support the findings of this study are available on request from the corresponding author.
